# Critical Assessment of a Structure-Based Pipeline for Targeting the Long Noncoding RNA MALAT1

**DOI:** 10.1021/acs.jcim.5c02955

**Published:** 2026-03-19

**Authors:** Riccardo Aguti, Mattia Bernetti, Gian Marco Elisi, Andrea Cavalli, Matteo Masetti

**Affiliations:** † Department of Pharmacy and Biotechnology, Alma Mater Studiorum, Università di Bologna, 40129 Bologna, Italy; ‡ Computational and Chemical Biology, Istituto Italiano di Tecnologia, 16163 Genova, Italy; § Department of Biomolecular Sciences (DISB),Università degli Studi di Urbino “Carlo Bo”, 61029 Urbino, Italy; ∥ Centre Européen de Calcul Atomique et Moléculaire (CECAM), Ecole Polytechnique Fédérale de Lausanne, 1015 Lausanne, Switzerland

## Abstract

Long noncoding RNAs (lncRNAs) are increasingly recognized as druggable targets due to their conserved secondary/tertiary structures and regulatory roles in disease. A prototypical example is the MALAT1 triple helix, whose stability supports transcript persistence and is implicated in oncogenesis. Here, we evaluate the ability of a structure-based drug discovery (SBDD) pipeline, integrating molecular dynamics (MD), pocket analysis, ensemble docking, and diverse scoring functions, to capture the binding behavior of 21 congeneric diminazene-based ligands targeting MALAT1. Conformational ensembles were generated using both conventional MD and Hamiltonian Replica Exchange MD, revealing two potential binding sites. Ensemble docking with AutoDock GPU and rDock across representative RNA conformations, followed by rescoring with force-field and machine-learning-based scoring functions, led to the identification of a binding mode with the best agreement across the series. Principal component analysis of interaction fingerprints within clustered poses was used to explain the experimentally observed affinity trends. Our findings highlight the promise and limitations of current SBDD pipelines for flexible RNA targets and offer a benchmark for future improvement in RNA-focused drug discovery.

## Graphical abstract

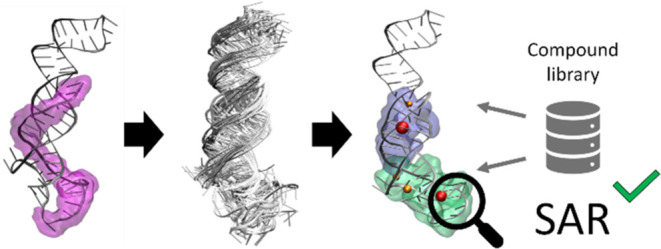

## Introduction

The organization of the eukaryotic genome is highly complex, with nearly 98% of the human genome consisting of noncoding sequences.^1,2^ Once dismissed as “junk DNA”, these regions are now recognized as a reservoir of functional elements, including multiple classes of noncoding RNAs (ncRNAs).^3–6^ Long noncoding RNAs (lncRNAs), which exceed 200 nucleotides, represent a highly heterogeneous class of regulatory molecules.^7–11^ lncRNAs often display greater conservation of secondary and tertiary structure than primary sequence,^12^ highlighting their functional relevance but also complicating experimental structure determination even with emerging techniques such as Cryo-EM.^13^ As the most abundant class of ncRNAs, lncRNAs participate in essential biological processes such as epigenetic regulation, transcriptional control,^14^ X chromosome inactivation,^15,16^ genomic imprinting,^17,18^ and cell development,^19^ and their dysregulation is strongly associated with disease, particularly cancer.^20^

A key example of the pharmacological relevance of lncRNAs is the metastasis-associated lung adenocarcinoma transcript 1 (MALAT1).^21^ Its overexpression has been reported across multiple cancer types,^22^ and its knockdown reduces oncogenic phenotypes.^23–27^ In particular, MALAT1 plays important roles in metastatic processes^23,24,26,28^ and in the development of resistance to several chemotherapeutic agents,^29–31^ thereby contributing to poor clinical outcomes. Despite extensive study, the molecular mechanisms and interaction partners underlying MALAT1-mediated regulation remain incompletely understood.^32^ MALAT1 is an attractive target for small-molecule intervention due to its well-characterized 3′-terminal triple helix, which is essential for transcript stability (Figure 1A).^33–38^ This motif, functionally characterized in cell-based assays^39^ and resolved through X-ray crystallography,^40^ protects the RNA from degradation by sequestering its adenine-rich 3′-tail via base pairing with a uridine-rich stem loop. Recent studies further indicate that local conformational dynamics modulate this protective mechanism, as increased flexibility or unfavorable ionic conditions destabilize the triplex, exposing MALAT1 to exonucleolytic degradation.^41^ Because small molecules can reshape RNA stability and conformational preference,^42–44^ this structural element represents a compelling therapeutic entry point.

**Figure 1:**
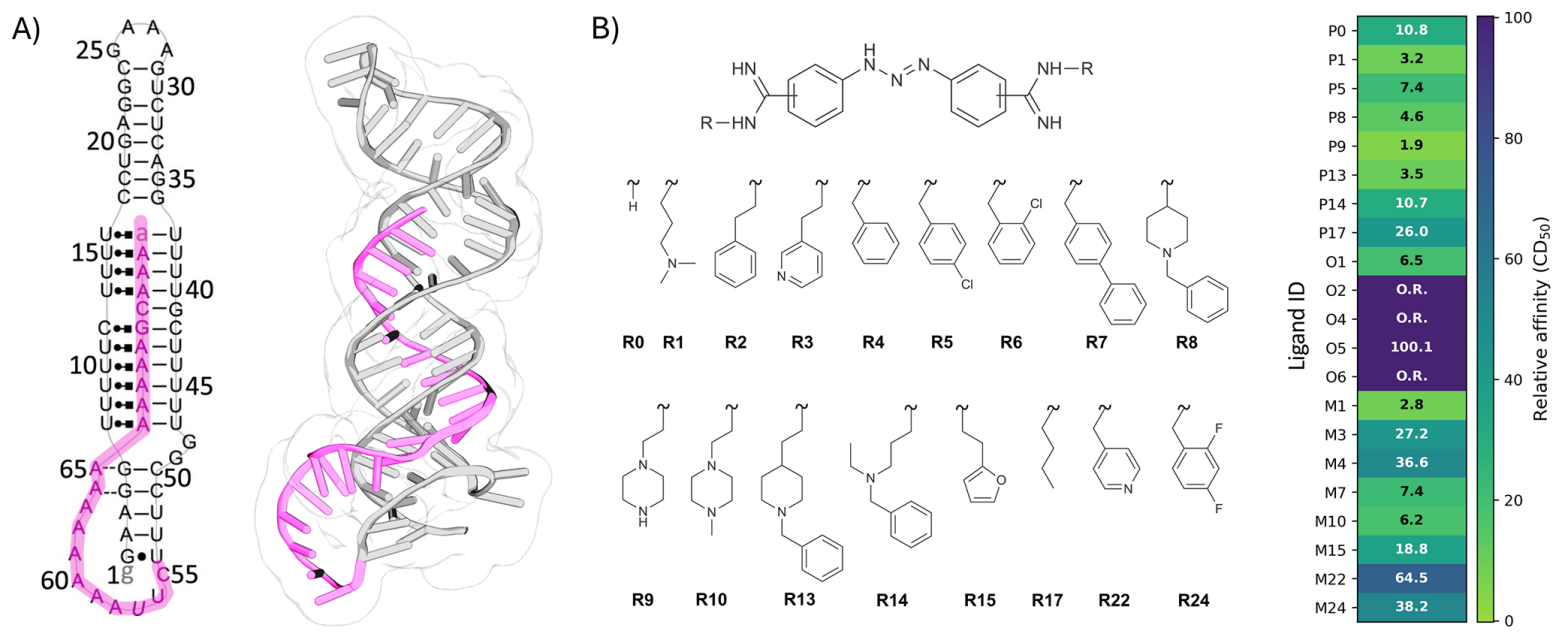
MALAT1 RNA triple helix and the diminazene series of ligands. (A) Secondary (left) and tertiary (right, PDB ID: 4PLX) structure of the MALAT1 triplex, with the poly­(A) tail highlighted in light purple. (B) The data set of small molecule ligands with diminazene scaffold used in this work, and corresponding experimental relative binding affinities in μM (right panel). Relative affinity is expressed as CD_50_ (small molecule concentration needed to achieve 50% competitive displacement); molecules labeled as out of range (O.R.) indicate small molecules with CD_50_ values outside of the range that lead to minimal dye displacement in the assays (>500 μM). The position of the substituents on the central scaffold (ortho, O, meta, M, or para, P), and the ID of the substituent compose the ligand ID.

Structure-based drug discovery (SBDD) is widely used to accelerate the identification and optimization of small-molecule ligands.^45^ However, most computational SBDD tools were developed for proteins, reflecting the relatively recent recognition of RNA as a druggable target.^46–48^ As a result, RNA-focused applications remain comparatively underexplored.^37,38,47–58^ Benchmarking studies have produced mixed conclusions: while RNA-specific tools such as rDock can outperform protein-oriented programs in pose prediction,^55^ broader analysis often favor protein-derived methods, including AutoDock GPU and AutoDock VINA among others, in overall performances.^56^ Moreover, no docking protocol has shown consistent success across enrichment metrics in RNA virtual screening campaigns.^57^ Crucially, these benchmarking studies have largely overlooked the challenge of generating binding-competent RNA conformations and identifying transient ligandable pockets. The recent disclosure of a library of 21 congeneric diminazene-based ligands targeting the MALAT1 triple helix, along with their experimentally measured affinities (Figure 1B),^59^ provides an ideal benchmark for evaluating current SBDD strategies against RNA targets. MALAT1 thus exemplifies the complexity of a real-world scenario, characterized by pronounced conformational heterogeneity and an unknown ligand binding mode and site.

In this work, we evaluate the applicability of SBDD frameworks based on molecular docking and Molecular Dynamics (MD) simulations to the MALAT1 triplex (Figure 2). We employ conventional MD^60^ and Hamiltonian Replica Exchange (HREX)-MD^61^ to extensively explore the RNA’s conformational space^62–64^ and identify potential ligand binding sites. Ensemble docking of the 21-ligand library is then performed against representative MALAT1 conformations using AutoDock GPU^65^ and rDock.^66^ Finally, we evaluate docking poses using force field- (AutoDock,^67^ AutoDock Vina,^68^ rDock^66^) and machine learning-based (AnnapuRNA,^69^ SPRank^70^) scoring functions to determine the ability of each protocol to recapitulate the experimentally observed variation in binding affinity and binding mode consistency across the library. This integrated approach reveals two persistent and ligand-accessible binding sites, one of which featuring a highly consistent binding mode across the ligand series, providing insight into affinity trends. Despite limited scoring accuracy, our results uncover meaningful structure–affinity trends and highlight key challenges and opportunities in RNA-targeted drug discovery.

**Figure 2:**
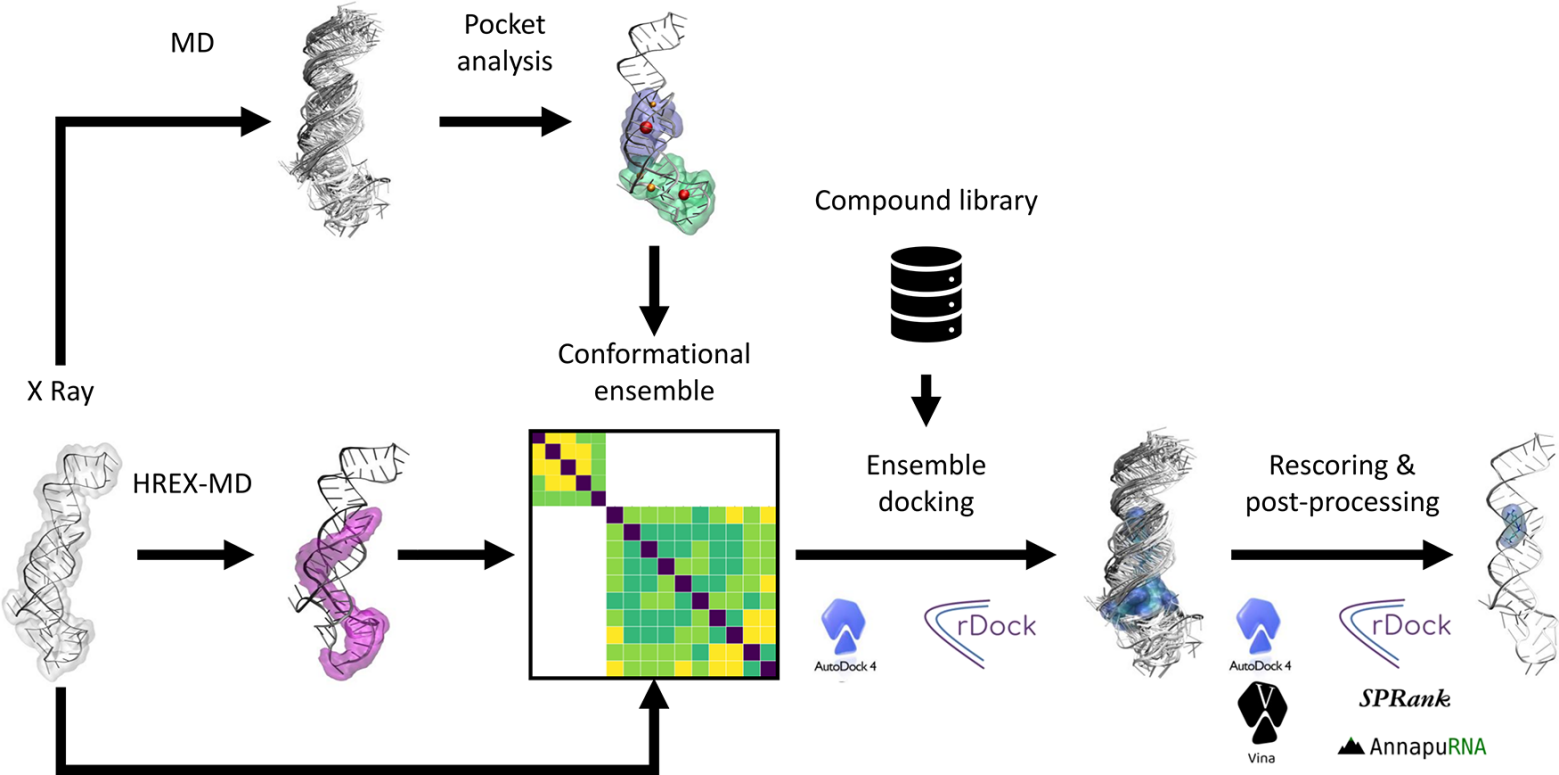
Computational pipeline established in this work. Starting from the crystal structure, standard and advanced MD simulations are employed to identify promising binding regions and to explore the RNA conformational dynamics, respectively. The information is integrated to generate a conformational ensemble of the identified binding regions, which is then used for ensemble docking of the diminazene library of compounds; ligand poses at this stage are produced using the AutoDock 4 and rDock software. Finally, the binding poses are rescored using diverse scoring functions (AutoDock 4, rDock, Vina, SPRank, and AnnapuRNA), and postprocessing of the outcomes allows rationalization of the results.

## Methods

### System Preparation

To perform molecular dynamics (MD) simulations, the RNA target structure (PDB ID: 4PLX)^40^ was modeled using the Amber force field for nucleic acids, comprising the Amber f99SB force field^71^ and the bsc0 ^72^ and χOL3 refinements.^73^

The system was solvated using the four-point OPC water model,^74^ while potassium and chloride ions were added to neutralize the system and achieve a physiological salt concentration of 150 mM KCl using the Joung and Cheatham ion model.^75^ While these conditions differ from those employed in the indicator displacement assay to determine ligand affinities (52 mM KCl and 0.1 mM MgCl_2_),^59^ the ionic concentration was chosen to approximate physiological conditions and thus promote the sampling of biologically relevant RNA conformational states. Magnesium ions were not explicitly included in our simulations due to the limited size of the simulation box. Subsequently, the system underwent energy minimization followed by equilibration in both the NVT and NPT ensembles, with a total simulation time of 1.2 ns (600 ps for each ensemble) with a time step of 2 fs. Covalent bonds involving hydrogen atoms were constrained using the LINCS algorithm,^76,77^ while long-range electrostatic interactions were treated with the Particle Mesh Ewald (PME) method,^78,79^ using a cutoff distance of 1.2 nm.

For conventional MD simulations in the NPT ensemble, the final equilibrated system was used as the starting point. For Hamiltonian Replica Exchange (HREX) simulations,^61^ the trajectory frame with volume closest to the mean from the second half of the NPT equilibration was used as initial configuration for production in the NVT ensemble. This choice mitigated artifacts from box size fluctuations that could lead to abnormal pressure values during subsequent HREX MD simulations performed under NVT conditions.

### Molecular Dynamics Simulations

Following system preparation and equilibration, production MD simulations were conducted on the MALAT1 RNA system, namely conventional MD and HREX MD.^61^ Conventional MD simulations were run in the NPT ensemble at 300 K and 1 bar, maintained by the V-rescale thermostat^80^ and C-rescale barostat^,^ ^81^ respectively. Three independent replicates, each lasting 500 ns long, were performed.

To enhance the conformational sampling of the RNA molecule, we employed the HREX scheme, which involves simulating multiple replicas of the systems with modified Hamiltonians where charges, Lennard–Jones parameters, and dihedral potentials of a predefined region of the system are gradually scaled.^61^ In this study, 16 replicas were used with the scaling parameter λ ranging from 1 to 0.7, applied specifically to residues 55 to 76 of the poly­(A) in the triple helix (Figure 1A). HREX simulations were run in the NVT ensemble at 300 K using the V-rescale thermostat.^80^ Each replica was simulated for approximately 96 ns, with exchange attempts every 240 steps. The trajectory from the “cold” replica (λ = 1) was extracted and processed for further analysis.

The conformational variability of the poly­(A) tail of the triple helix was inspected in both the conventional and HREX MD simulations, focusing on the “coldest” replica in the latter case, by computing the RMSD, aligning and computing on the RNA heavy atoms, and eRMSD, based on nucleotide g-vectors, with respect to the starting structure.^82^ While the RMSD measures the overall structural deviation, the eRMSD is specifically designed for RNA and reflects base-pairing rearrangements, with values above 0.7–0.8 typically indicating discrepancy in the base-pairing pattern with respect to the reference.^82^.

### Pocket Identification

The three trajectories from conventional MD of the RNA were aggregated and analyzed with the Pocketron algorithm to identify dynamic pocket formation and communication.^83,84^ Pocketron, included in the BiKi Life Sciences software package,^85^ tracks the dynamic evolution of binding pockets during MD simulations, using the NanoShaper^86,87^ algorithm to detect pockets on a frame-by-frame basis. The approach leverages solvent-excluded surface calculations with two probe sizes, and we used here the default radii of 1.4 Å and 3.5 Å for the smaller and larger probes, respectively. To focus on pockets with potential relevance for small-molecule binding and long-range allosteric communication, only cavities larger than the volume of three water molecules were tracked. This filtering step excludes highly transient or solvent-exposed pockets that are unlikely to accommodate ligands. The analysis allows identifying pockets exhibiting the largest volume, highest persistence, and significant long-range communication.

### Conformational Ensemble Preparation

The HREX-MD simulation was analyzed to extract a representative ensemble of conformations for subsequent docking studies. The conformational variability of the regions identified as promising binding sites, based on the pocket analysis on the conventional MD runs, was evaluated in the “coldest” replica (λ = 1) of the HREX. We first determined the RMSD, aligning and computing on the RNA heavy atoms, and eRMSD, based on nucleotide g-vectors, with respect to the starting structure.^82^

Principal Component Analysis (PCA) was performed via the scikit-learn Python package,^88^ using as input features the g-vectors of residues comprised in the two selected binding regions. Finally, to identify representative conformations of the two sites from the HREX-MD simulation, we applied the Quality Threshold (QT) clustering algorithm^89^ to the eRMSD matrix of the frames from the coldest replica, using a cutoff of 0.7 to group similar structures and extracting centroids from each of the identified cluster. We then retained cluster centroids with eRMSD > 0.7 with respect to all other cluster centroids in order to maximize the structural variability of the ensembles. The procedure was repeated separately for the two binding regions identified.

### Docking Pose Generation

Docked poses of the 21 diminazene analogs were generated using two software packages, namely AutoDock GPU^65^ and rDock, with default settings.^66^ Both software require dedicated preparation of the biomolecular target and the ligands, using a united-atom representation with explicit hydrogens on polar heavy atoms only. A notable difference between the two programs is the grid definition for docking. In both software, the grid was centered on the center of mass of the heavy atoms of the residues comprised in the binding sites. In AutoDock GPU, a rectangular grid needs to be defined (Figure S1), for which we employed 110 × 110 × 130 points for the first site and 110 × 80 × 80 points for the second one, with a grid spacing of 0.3 Å between points. These dimensions were chosen to ensure complete coverage of the binding regions identified through Pocketron. Differently, rDock employs a two-sphere approach for grid generation. Here, we used a radius of 17 Å for both sites, with a small probe of 1 Å and a large probe of 17 Å. This aimed at creating a spherical grid encompassing about the dimensions of the AutoDock one, to ensure comparable volumes allowed for pose generation via the two docking software. For each of the 21 ligands, we generated 250 poses with each software.

### Pose Rescoring

To evaluate the generated ligand binding poses on the RNA target, we applied multiple scoring functions, encompassing both force field-based and machine learning-based approaches. Specifically, as force-field-based methods, we employed the scoring functions from AutoDock,^67^ rDock,^66^ and Vina.^68^ On the machine learning side, we used the recently developed AnnapuRNA^69^ and SPRank^70^ scoring functions. AnnapuRNA leverages a coarse-grained representation of RNA and ligand pharmacophores, which is used to determine RNA-ligand interaction scores using a trained ML model. SPRank refines knowledge-based pairwise potentials iteratively against experimental RNA-ligand structures to discriminate correct poses.

The Vina, AnnapuRNA, and SPRank scoring functions can be directly applied to the output poses from AutoDock GPU and rDock after minor formatting conversion. However, artifacts arose when rescoring some of the poses generated by rDock with the AutoDock GPU scoring function, evidenced by high score values. To address this issue, the grid box for both binding regions was extended to 130 × 130 × 130 points in the three dimensions during the rescoring phase. For consistency, also the poses generated by AutoDock GPU were rescored using this grid definition.

### Interaction Fingerprints

To characterize RNA-ligand interactions in the generated binding poses, we computed interaction fingerprints via the fingeRNAt^90^ Python package. The tool analyzes complexes between nucleic acids and ligands to generate binary vectors indicating presence or absence of specific noncovalent interactions, namely hydrogen bonds, halogen bonds, lipophilic interactions, cation–anion, π–stacking, π–cation, and π–anion. The software inspects the residues in nucleic acid structures using geometric criteria, and the analysis supports three modes: SIMPLE, for detecting basic atomic contacts; PBS, for distinguishing interactions involving phosphate, base, and sugar moieties of nucleic acids; FULL, for classifying the interactions into specific categories. Herein, we applied fingeRNAt to the RNA-ligand binding poses using the FULL method. For every pose, histograms of interaction occurrences were computed at distances ranging from 2 to 8 Å with 80 bins. Additionally, PCA was applied on the interaction fingerprints, using bin populations as input features.

### Clustering of the Binding Poses

Cluster analysis of the binding poses was achieved through the Quality Threshold (QT) clustering algorithm^89^ applied to the minimum distance RMSD matrix^91^ with a threshold of 5 Å. The RMSD matrix was computed focusing on the common diminazene scaffold of the 21 compounds. For proper clustering of the generated ligand binding poses, structural symmetry must be taken into account. To this end, RMSD values between pose pairs were computed on the Cartesian coordinates of the ligand scaffold both directly and after applying symmetry transformations,^92^ and the minimum RMSD value was retained. This was particularly important for para-substituted ligands with symmetrical side chains (Figure S2).

### Stability of Docking Poses

Standard MD simulations were performed on the docking complexes of compounds O5, P0, and P9 from cluster 3 of Site 2. The systems were parametrized with the OPLS4 force field^93^ and minimized with MacroModel,^94^ treating the ligands and the surrounding nucleic bases within 5 Å as flexible. The complexes were solvated in explicit TIP3P water using orthorhombic simulation boxes. Potassium and chloride ions were added to neutralize the systems and achieve salt concentrations of 52 and 150 mM. Following 100 ps of Brownian dynamics with positional restraints applied to the solute atoms, the systems were heated to 300 K in the NVT ensemble over 1 ns. This was followed by a gradual release of restraints over 1.5 ns in the NPT ensemble. Prior to production, an additional 5 ns NPT equilibration stage was performed with weak positional restraints of 0.5 kcal mol^–1^ Å^–2^ applied to the phosphate atoms and ligand heavy atoms. Production runs were carried out in the NPT ensemble without restraints, using the Nosé-Hoover thermostat^95,96^ and Martyna–Tuckerman–Klein barostat.^97^ For each ligand and ionic condition, five independent replicates of 200 ns each were performed, resulting in 30 MD runs for an aggregate total of 6 μs. Covalent bonds involving hydrogen atoms were constrained using the M-SHAKE algorithm.^98^ Short-range electrostatic interactions were truncated at 9 Å, whereas long-range electrostatics were treated using the PME method.^79^ A RESPA integrator^99^ was employed with a 2 fs time step for bonded and short-range nonbonded interactions, while long-range electrostatics were updated every 6 fs. All simulations were performed using Desmond v8.2.^100^

## Results and Discussion

### Sampling the Conformational Space of the MALAT1 Triple Helix

Generating an ensemble of drug-target conformations by MD simulations for subsequent virtual screening campaigns is a well-established SBDD approach.^101^ Motivated by the need to explore MALAT1’s conformational space and identify suitable binding pockets, we carried out simulations starting from the available X-ray structure (PDB ID: 4PLX).^40^ Three independent conventional MD simulations alongside a single HREX-MD simulation were performed. While conventional MD simulations can be limited in their ability to efficiently sample the complex conformational landscape of biomolecular systems,^102^ this limitation is expected to be particularly pronounced for RNA targets due to their intrinsic flexibility.^62,64,103^ Following this rationale, and within a realistic-case scenario in which binding sites and their degree of preorganization are not known a priori, HREX-MD was employed under the assumption that an enhanced sampling protocol would enable a more exhaustive exploration of the conformational space, thereby improving the detection and characterization of transient and alternative binding pockets. The triplicate conventional simulations then served as a baseline for quantifying the benefit of this enhanced sampling protocol.

Figure 3A illustrates the conformational dynamics of the poly­(A) tail under conventional MD sampling. In particular, the RMSD analysis reveals significant structural fluctuations, especially in Run 1, where RMSD values reach up to 8 Å. In contrast, the eRMSD timeseries remain more consistent across all replicates, with values centered around 0.7, and only occasionally approaching 1 in Run 3. This suggests that the high RMSD values observed do not necessarily correspond to significant base pairing rearrangements, emphasizing the importance of eRMSD as a more tailored metric for capturing relative changes in nucleic acid structure. Furthermore, the PCA plot, color-coded by replica, shows that even relatively short simulations access a distinct conformational space in the three runs. As a comparison, Figure 3B demonstrates the effectiveness of HREX-MD in sampling the conformational landscape of MALAT1 more exhaustively, as evidenced by the corresponding eRMSD timeseries, reaching values up to 1.2. The improved sampling can be better appreciated through dimensionality reduction via PCA (Figure 3C), which shows that the HREX-MD not only fully covers the PCA space spanned by the three conventional MD runs but also explores previously uncharted regions. By analyzing and visually comparing the centroid structures, we observe that, compared with the conventional MD, the HREX simulations promote greater mobility of the poly­(A) residues, particularly in the lower region where the triple helix wraps around the duplex and inserts to form the triple pairing. In this region, where the residues are less constrained, larger displacements are detected due to lost interactions involving particularly residues U56–U57, A60–A61, and U52–U53. This enhanced flexibility also reveals a new cluster of conformations sampled only in the HREX simulations, in which the lower region folds back on itself, bringing residues A60 and A61 into close proximity with U52 and U53, while displacing U56 and U57 away from the duplex. In contrast, in the central region where the triple pairing occurs, no major differences are observed compared with the conventional MD simulations, apart from minor local shifts.

**Figure 3:**
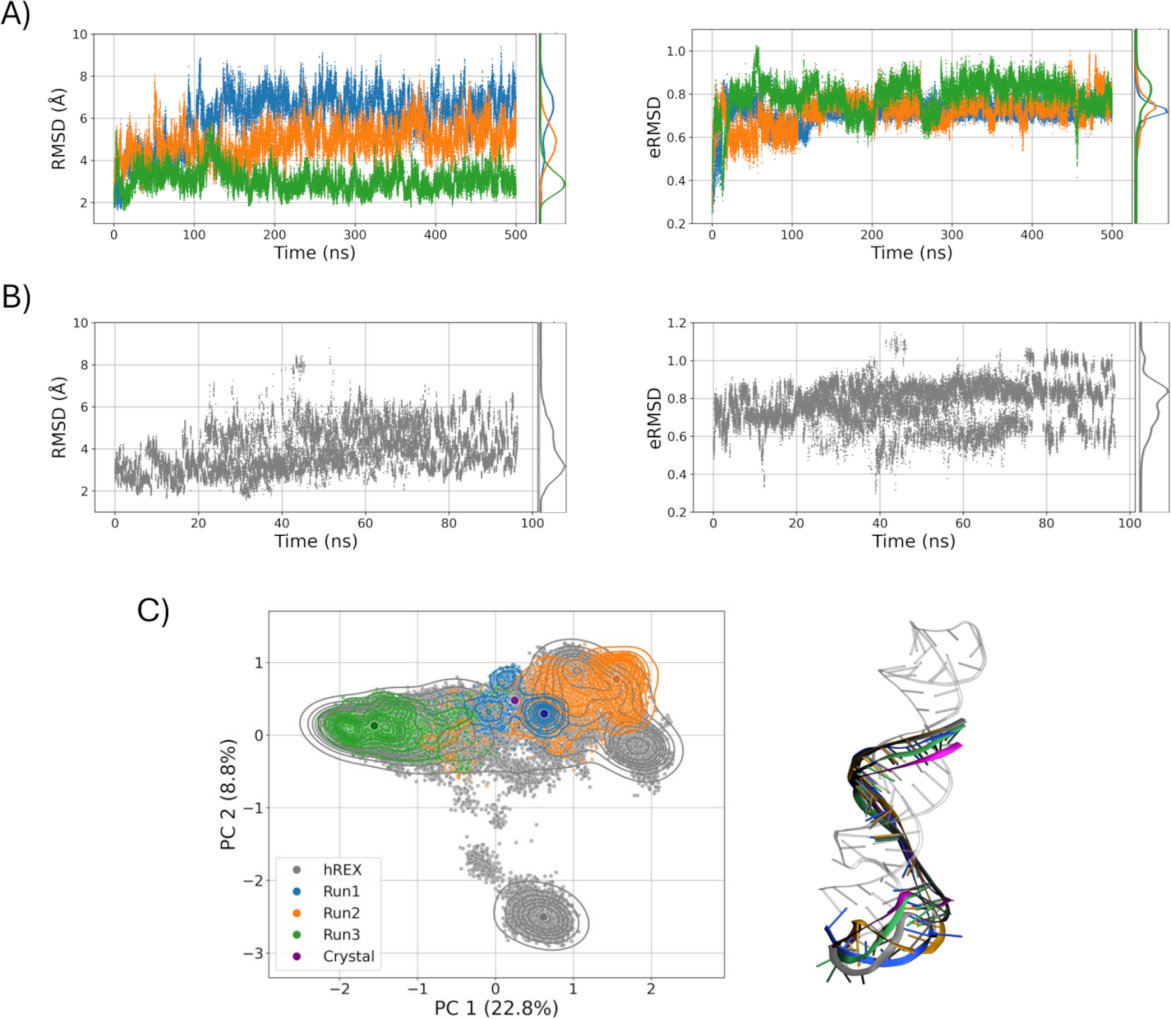
Conformational dynamics of MALAT1 in conventional and enhanced MD simulations. Timeseries and distribution of RMSD and eRMSD computed on the poly­(A) residues using the crystallographic conformation as a reference in (A) three independent conventional MD simulations and (B) the HREX MD simulation. (C) PCA of the aggregated conformations sampled in the conventional and HREX MD simulations, color-coded consistently with panels (A, B) and with the crystallographic conformation indicated in purple. The circles indicate the representative conformations of the poly­(A) tail from the most populated clusters of each simulation. On the right, the representative conformations are superimposed on the crystal one using the same color scheme.

### Pocket Communication Analysis Reveals Potential Binding Sites

Sampling a broad conformational space of the MALAT1 triple helix does not necessarily imply the presence of binding sites capable of accommodating the small molecules in the reference library, nor does it guarantee that the observed conformational variability is reflected in pocket diversity. To address these issues, we carried out a systematic pocket identification and pocket communication analysis using the Pocketron algorithm. The analysis yielded a total of 37 pockets, encompassing those present in the initial structure and those dynamically formed during the simulation. A comprehensive picture of pocket communication within the MALAT1 structure is reported by the 37 × 37 correlation matrix derived from the Pocketron analysis (Figure S3), where each element quantifies the communication between corresponding pocket IDs (pIDs). These pockets can be visually represented as spheres (Figure 4A), with sphere radius proportional to pocket volume and color indicating pocket persistency (percentage of trajectory frames with nonzero volume).

**Figure 4:**
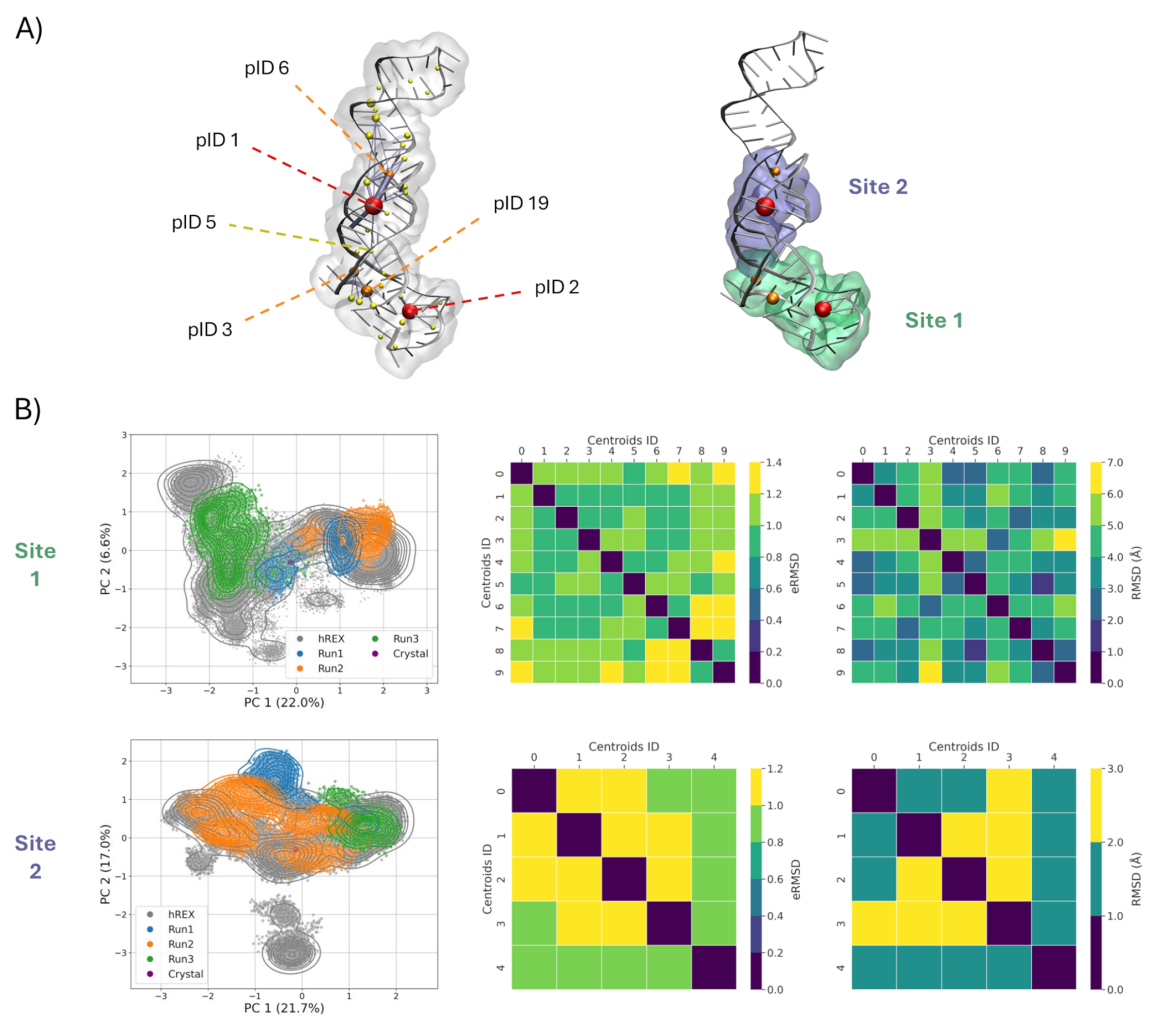
Generating a conformational ensemble of the promising binding regions. (A) Pocket communication analysis on the standard MD simulations (left) identified two major interconnected regions in the MALAT1 structure, to use as promising binding sites for subsequent docking (right). Yellow, orange, and red spheres correspond to pockets displaying increasing persistence, with sphere size reflecting the pocket volume, and edge thickness proportional to communication strength. (B) PCA of the combined MD simulations (left), and eRMSD and RMSD matrices of most dissimilar cluster centroids from the HREX MD simulations (right), performed separately for the two binding regions.

The color scheme corresponds to the following persistence ranges: yellow (<33%), orange (33–66%), and red (>66%). Notably, the most persistent pockets also exhibit larger volumes, suggesting their potential suitability for further analysis and targeting in drug discovery efforts. To further assess the suitability of these pockets for ligand binding, we employed Pocketron to track residue exchanges between pockets and quantify the extent of communication across different target regions. This involved calculating the average number of “merge” and “split” events,^84,104^ which can be then visualized in a 3D graph (Figure 4A), with edge thickness proportional to the strength of communication between pockets. Overall, the analysis reveals two promising regions that harbor the largest and most persistent pockets. In the lower portion of the triple helix, a localized exchange of residues around the high-volume, high-persistence pocket 2 can be observed. In the adjacent region, another short communication pathway is present, including pockets 19 and 3, displaying a slightly lower persistence. Interestingly, a more long-range pathway originating from pocket 19 can also be observed, traversing pockets 3 and 5 to reach the second high-volume, high-persistence pocket 1, in the upper portion of the structure. Based on these insights, we next focused our analysis on the two regions comprising the most persistent pockets (Figure 4A and Table 1). These regions, hereafter named Site 1 (including pIDs 2, 3, and 19, in the lower portion of the triple helix) and Site 2 (including pIDs 1 and 6, in the upper portion), represent promising areas for further exploration as ligand binding sites, and consistent with those previously explored in independent studies. In particular, our Site 1 corresponds to site 3 in François-Moutal et al.^33^ and comprises the site explored in the works by Rocca et al.^37,38^ and the one bound by compound 16 in Abulwerdi et al.,^34^ while our Site 2 corresponds to site 2 in François-Moutal^33^ and to the site bound by compound 5 in Abulwerdi et al.^34^ Notably, not only do the pockets engage in frequent residue exchanges with neighboring ones, but they also exhibit communication across the two regions, albeit to a lesser extent. This is interesting, as intersite communication hints at potential allosteric effects^105^ that could propagate through the structure and ultimately influence the stability of the entire triple helix.

**Table 1:** Features of the Major Pockets Identified from the MD Simulations, Namely Pocket ID, Average Volume, Residues Comprised in the Pocket, Pocket Persistency, and Corresponding Binding Region

pID	average volume (Å^3^)	residues	persistency (%)	site
2	297	3, 4, 5, 6, 52, 53, 54, 56, 57, 58, 59, 60, 61, 62, 63, 64	69	1
3	179	47, 49, 50, 66, 67	62	
19	294	49, 50, 51, 52	35	
				
1	455	11, 12, 13, 41, 42, 70, 71, 72	96	2
6	99	13, 14, 15, 73, 74	42	

### Characterization of a Site-Specific Conformational Ensemble

With the binding site definition established, we next characterized the conformational ensemble spanned by Site 1 and Site 2 in terms of the spatial arrangements of their constituent residues. In analogy with the previous analyses, we computed site-specific eRMSD and performed PCA (see Methods section) for both the conventional and HREX MD trajectories. Figure S4 illustrates the eRMSD time series for both sites, with values ranging from approximately 0.6 to 1.3. HREX-MD simulations expanded the conformational space accessible to the two regions beyond that sampled by the three independent conventional MD runs. This effect is particularly evident in the PCA analysis (Figure 4B, left panels). Comparing the two sites, Site 1 maintained a base pairing pattern closer to the reference compared to Site 2 (Figure S4), whose dynamics were mostly driven by rearrangements of the triple helix. Specifically, Site 1 spanned values between 0.7 and 1.1, whereas Site 2 explored higher eRMSD values, except for Run 3, which remained closer to the reference.

To identify representative conformations of the two sites, we performed cluster analysis. To this end, we applied the Quality Threshold clustering algorithm^89^ to the eRMSD matrix of the residues comprised in the two regions. The overall conformational diversity is captured by the pairwise eRMSD matrices (Figure 4B, center and right panels), with eRMSD values spanning the 0.7–1.4 range for Site 1 and 0.7–1.2 for Site 2, highlighting a greater conformational variability, and thus greater flexibility, for Site 1. Then, to maximize the structural variability of the representative structures, we only retained cluster centroids with eRMSD above the cutoff of 0.7. This resulted in two independent ensembles comprising 10 and 5 structures for Sites 1 and 2, respectively. Taken together, these curated ensembles captured the full range of conformational dynamics observed in the simulations and provide a minimal, yet representative, set of structures to support efficient downstream docking studies.

### Complementary Binding Modes by Distinct Docking Programs

In a recent work, a library of 21 congeneric compounds was experimentally tested against the MALAT1 triple helix, providing a reference set of small molecules.^59^ The compounds share a diminazene scaffold, decorated with different substituents in the ortho, meta, and para positions (Figure 1B). Thus, we used the generated conformational ensembles for the two binding regions in the RNA to dock the 21 compounds, in an ensemble docking spirit.^47,106^ We also included the conformations of Sites 1 and 2 of the crystal structure of the RNA, yielding 17 combined conformations. Using AutoDock GPU and rDock, we generated 250 poses per ligand with each program (500 poses per ligand overall). This process was repeated for all representative structures of the two sites, resulting in 8500 docked poses for each compound. We first assessed the extent of agreement between the poses generated by the two programs by performing dimensionality reduction across all the poses for all the RNA target conformations within each site.

Figure 5 shows the results of PCA on the Cartesian coordinates for 5500 poses across 11 RNA conformations for Site 1 and 3000 poses across 6 RNA conformations for Site 2, obtained with the two software for compound P13, which has the largest number of heavy atoms and represents the most challenging case for accommodation within the target due to its marked steric hindrance. While the poses occupy overlapping regions in the PC space, rDock was able to generate binding modes not accessed by AutoDock GPU, particularly in Site1. This result underscores the value of using multiple docking programs to capture a broader spectrum of potential binding poses on the RNA target. For the smaller Site 2, the poses from both programs occupy similar regions in the PC space, indicating lower diversity between the generated poses. Overall, for both sites, the first PC distinguishes elongated binding modes along the major groove and more compact ones.

**Figure 5:**
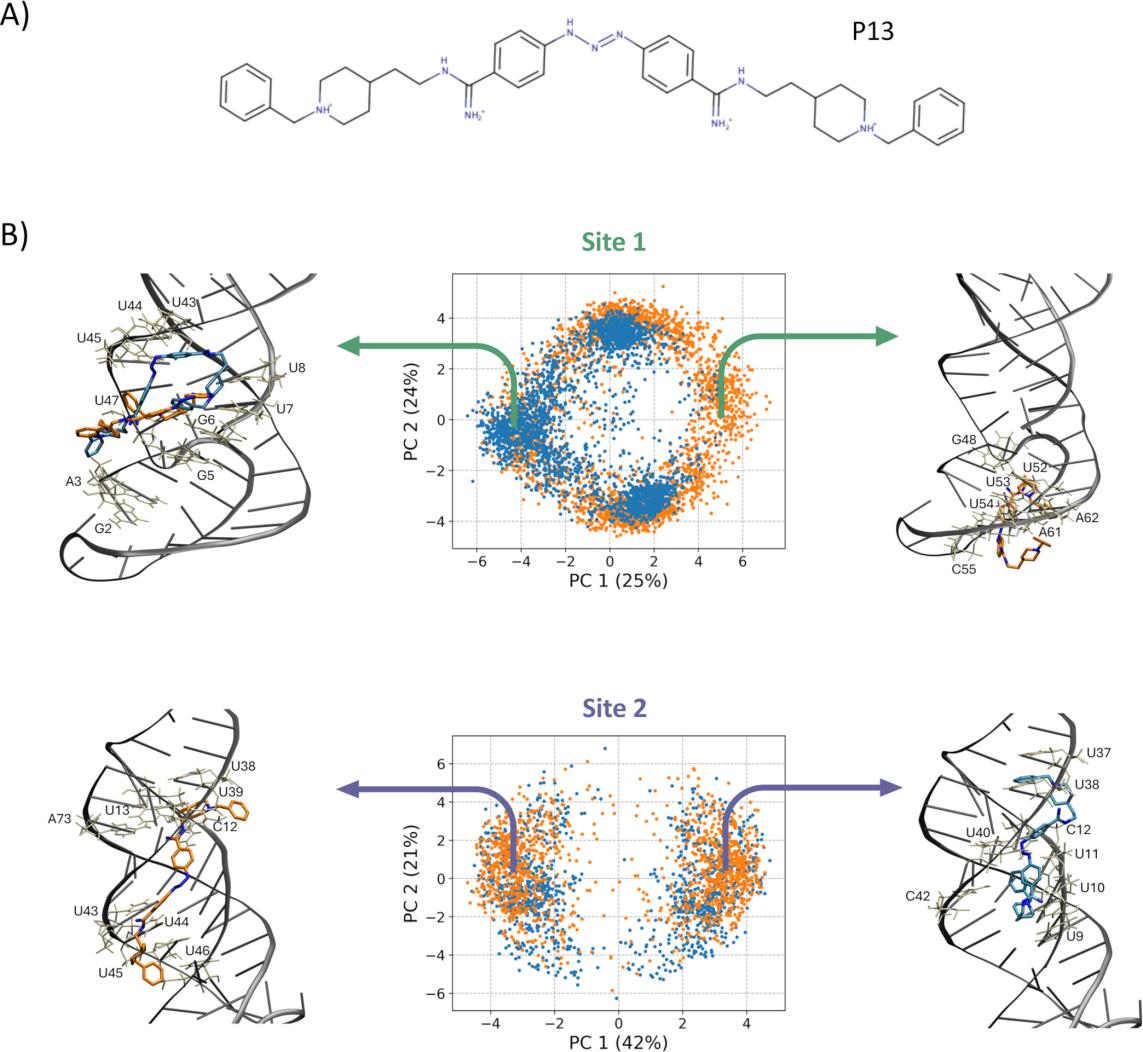
Binding poses generated for ligand P13. (A) 2D structure of the diminazene analog P13. (B) PCA on the aggregated poses generated for ligand P13 at the two sites, using the AutoDock GPU (blue) and rDock (orange) docking software. Representative binding poses from extreme regions along PC1 are shown, with ligand carbon atoms colored consistently with the software that generated the pose, while RNA residues within 3 Å of ligands are displayed in licorice, colored white, and labeled.

### Scoring Function Performance and Conformational Preference

To investigate how different scoring functions evaluate the same set of docked poses, we rescored all poses using multiple scoring functions, namely AutoDock, rDock, AutoDock Vina, AnnapuRNA, and SPRank. An overview of the results, covering all ligands and all combinations of docking programs and scoring functions for both Site 1 and Site 2, is reported in Figures S5–S8. The general trend that can be inferred is that poses tend to have more favorable scores when evaluated by the native scoring function of the program that generated them. For example, poses from AutoDock GPU scored overall better under the AutoDock scoring function, while poses from rDock performed better when evaluated by the native rDock scoring function (Figures S5 and S6). This effect is more pronounced for rDock, which shows greater variability across the scores. AutoDock Vina exhibited a similar pattern to AutoDock, although to a lesser extent, suggesting it identifies stable poses across both docking programs more uniformly. By contrast, AnnapuRNA showed lower consistency (Figures S7 and S8), with poses generated by both programs largely overlapping within the same score range. However, several poses produced by AutoDock GPU received highly unfavorable scores, likely due to steric clashes. Interestingly, this behavior was absent when scoring rDock-generated poses, suggesting higher compatibility between the RNA-specific docking program and this scoring function. Nevertheless, this trend could not be generalized to all considered RNA-specific scoring functions, since SPRank exhibited the opposite behavior, assigning more favorable scores to AutoDock GPU poses in both binding sites.

These patterns are further illustrated in Figure S9, which shows the top-scoring poses for each ligand across all docking program-scoring function combinations. AutoDock and rDock scoring functions consistently favored their respective docking outputs for both sites. AnnapuRNA favored rDock-generated poses, while Vina showed a site-dependent behavior. In particular, for Site 1, Vina generally preferred rDock poses, though a few AutoDock GPU poses were also highly ranked. Conversely, for Site 2, Vina leaned toward AutoDock GPU poses. Similarly, SPRank favored AutoDock GPU poses for Site 2, while it identified top-scoring poses from both AutoDock GPU and rDock for Site 1.

To assess the effect of target conformation on docking outcomes, we compared the top-ranked pose from each conformational ensemble for every ligand (Figure S10). Site 1 exhibited a broad distribution of best-scoring structures, with conformers 5, 7, 10, and the crystal structure frequently yielding the top scores across several scoring functions. AutoDock produced mixed results, favoring conformers 7, 10, and the crystal structure, whereas AutoDock Vina showed an even stronger preference for the crystal structure. rDock displayed a clear bias toward conformer 10, while ML-based scoring functions yielded more variable rankings: AnnapuRNA tended to favor conformers 5 and 10, and SPRank preferentially selected conformers 5 and 7. In contrast, Site 2 displayed more consistent behavior, with fewer conformers dominating (Figure S11). When considering the best score per ligand irrespective of the target conformation, both AutoDock and Vina most frequently favored conformers 1, 3, and the crystal structure, with Vina showing a marked preference for the latter even at Site 2. Conformer 4 consistently emerged as the preferred structure for rDock across all ligands, as well as for AnnapuRNA, though the latter occasionally favored conformer 1. SPRank generally preferred conformers 4 and 3, with minor deviations across individual ligands.

### Evaluation of Docking Scores against Experimental Affinity Trends

Our goal for the ensemble docking exercise was to evaluate whether any combination of docking software and scoring function could reproduce the experimentally observed affinity trends among the ligands. In this respect, the overall picture is summarized in Figure S11, which reveals that no clear correlation with experimental binding affinities could be established, except for AutoDock to a limited extent. For the latter, higher-affinity ligands generally received more favorable scores than lower-affinity ones, suggesting that AutoDock scores may be suitable in distinguishing binders from nonbinders.

Interestingly, different from other scoring functions, AutoDock results displayed highly similar scoring patterns across the ligand set for both binding sites. This implies that the AutoDock scoring is largely independent of the specific binding site, highlighting a potential limitation in its ability to capture site-specific interactions for this case. The comparable scoring behavior across sites may stem from the chemical composition of the two binding regions, both featuring A-U base pairs with two G-C pairs in the middle. This similarity in composition can be particularly relevant for force-field-based scoring functions such as AutoDock, which heavily rely on atomic partial charges and van der Waals interactions.

Although AutoDock showed the most pronounced site-independent behavior, similar but less marked scoring patterns emerged for Vina and SPRank. Particularly interesting is the similarity observed within Site 1 for AnnapuRNA and SPRank. This comparable trend indicates that certain physicochemical properties of the ligands themselves may drive scoring outcomes across different methods. Overall, compared to AutoDock, AnnapuRNA and SPRank preserve some degree of site-specificity in their evaluations.

### Interaction-Based Interpretation of Ligand Affinity

To rationalize the observed variation in experimental binding affinities based on the predicted binding modes, we focused on ligand poses generated by AutoDock GPU, as its scoring function was the only one to exhibit a discernible correlation with experimental trends, albeit with the limitations discussed above. In particular, we analyzed ligand interactions within Site 1 and Site 2 using the recently developed fingeRNAt.^90^ The tool inspects complexes between nucleic acids and ligands to detect and classify noncovalent interactions. All ligand poses across the conformational ensemble were evaluated to generate an average interaction profile. As shown in Figure S12, the average interaction profiles were broadly similar between the two sites due to their comparable chemical features. A notable exception involved lipophilic interactions: Site 2 showed a sharp peak at approximately 5 Å, while Site 1 exhibited a bimodal distribution with peaks around 4.5 Å and 5.5 Å. To detect dominant interaction patterns among ligand poses, we further applied PCA. Although differences between sites were subtle, a clear trend emerged along PC1. Specifically, poses derived from low-affinity ligands were predominantly located in the negative region of PC1, whereas those from high-affinity ligands clustered toward positive PC1 values, indicating a separation in interaction patterns consistent with binding strength. In particular, analysis of the PC loadings revealed that positive values along PC1 are mainly associated with hydrogen-bond (distance range 2.25–3.88 Å) and cation–anion (2.23–5.45 Å) interactions. By contrast, lipophilic contacts (2.75–3.95 Å) are primarily associated with poses located on the negative side of PC1. All other interactions and distance ranges display no significant contribution in the loading profile (Figure S13).

To improve the interpretability of results, we performed a cluster analysis on the ligand binding poses. To discriminate major configurations of the ligands, we focused on the common diminazene scaffold of the 21 compounds. For this stage, we only retained the most populated clusters, covering about 50% of poses for further analysis (Figure 6A). PCA of these selected clusters (Figure 6B) showed improved separation of ligands by affinity, and the variance explained by the first two principal components increased from about 14% to approximately 26%, underscoring the effectiveness of this refinement.

**Figure 6:**
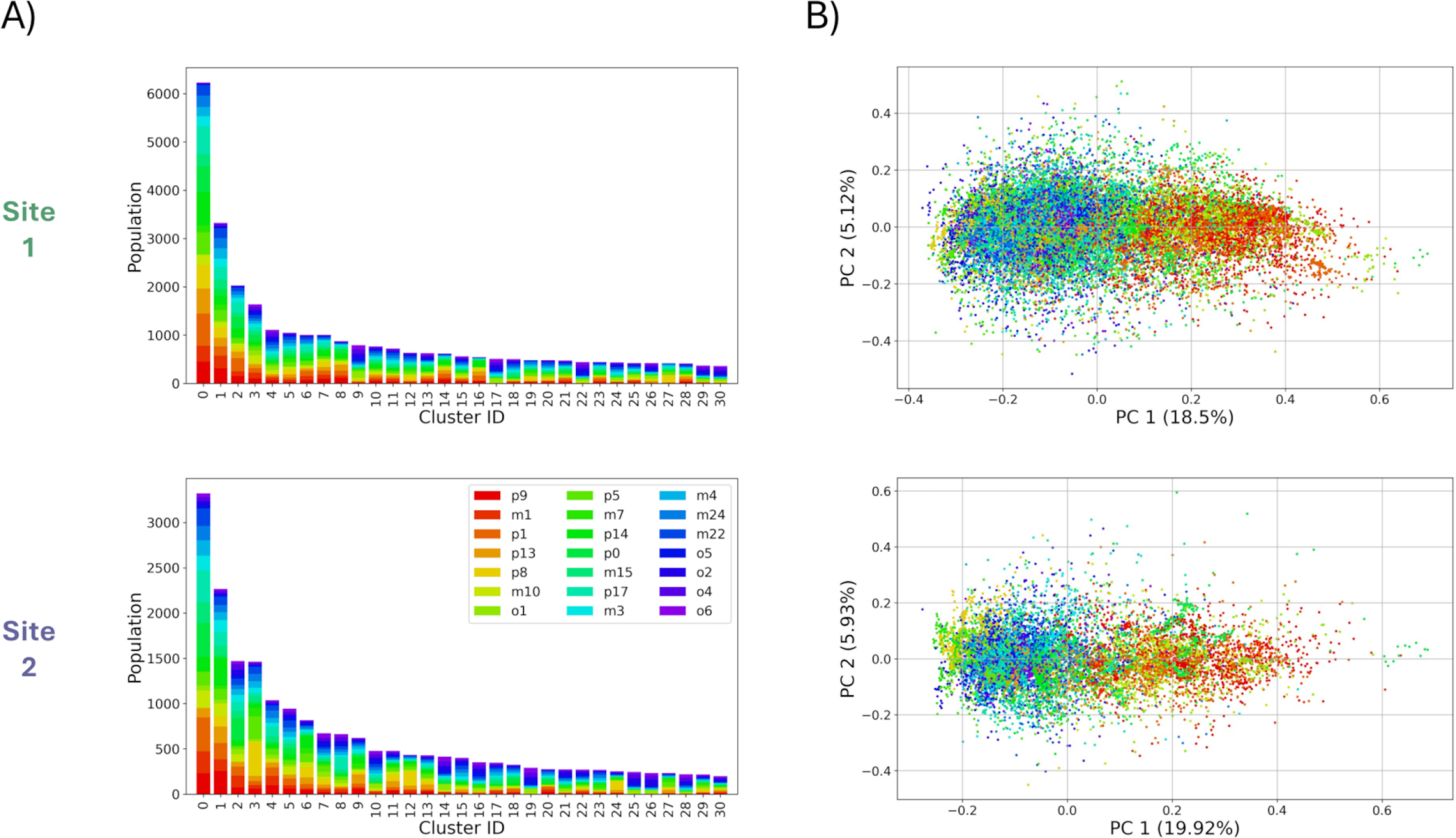
Characterization of the aggregated binding poses generated for all ligands. (A) Cluster analysis and (B) PCA were performed separately on Sites 1 and 2 (top and bottom panels, respectively), using about 50% of the total number of poses. Cluster populations are highlighted by occurrence per ligand, and the same color code is used for the PCA projections, ranging from red (highest relative affinity) to violet (lowest relative affinity).

To further explore affinity-related binding patterns, we analyzed the distribution of PC values within each cluster. For each ligand, we calculated the average PC1 score per cluster (Figures S14–S17). Cluster 3 of Site 2 showed the strongest association with experimental binding affinities (*R* ^2^ = 0.6, Figure 7A), suggesting that poses in this cluster, which feature a distinctive and consistent binding mode of the central scaffold, are most effective at distinguishing strong from weak binders.

A closer inspection of the binding poses within this cluster, focusing on ligands P9 (highest experimental affinity), P0 (unsubstituted scaffold), and O5 (lowest affinity), provides additional insight into the observed structure–activity relationships (Figure 7B). Starting with P0, which represents the common scaffold, the central benzene rings establish lipophilic contacts with the RNA bases, while the diminazene group forms hydrogen bonds with nearby residues. The amidine moiety at the opposite end further participates in hydrogen bonding with bases and ionic interactions with the phosphate backbone via its positively charged nitrogen atoms. The binding mode of P9 closely resembles that of P0 in terms of scaffold and amidine interactions. However, its nitrogen-rich, positively charged substituents enable additional hydrogen bonds and cation–anion contacts with the RNA phosphate groups. This expanded polar interaction network corresponds to the shift of P9 poses toward higher PC1 values in the PCA plot. By contrast, O5, which bears highly lipophilic substituents, predominantly forms lipophilic contacts with the aromatic bases, resulting in lower PC1 values. Overall, the experimental affinity trend can be rationalized based on the substituents’ charge and hydrogen-bonding capacity: nitrogen-rich, positively charged groups generally enhance affinity relative to P0, whereas purely aromatic or hydrophobic substituents reduce it, with the only exceptions being P5 and M7.

**Figure 7:**
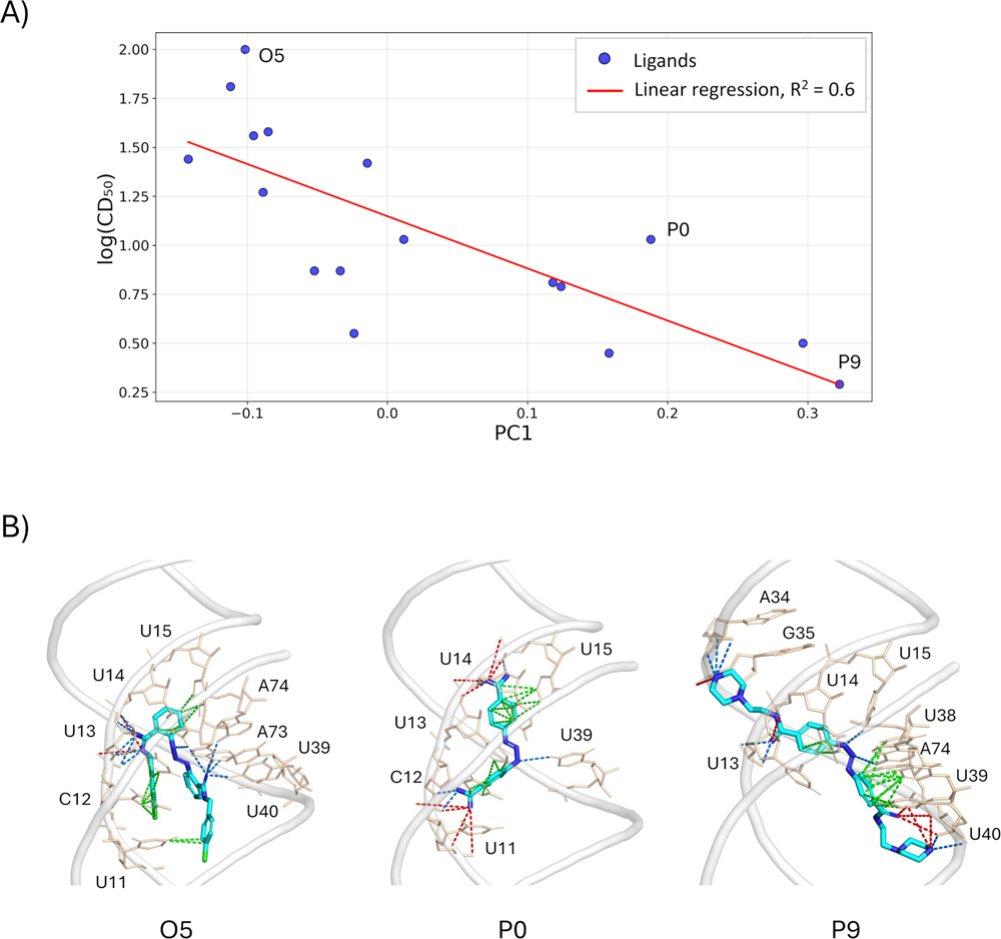
Rationalizing ligand affinity by predicted binding mode. (A) Linear relationship between relative affinity and average PC1 score for the entire set of compounds. (B) Proposed binding modes for representative ligands, namely the highest-affinity one (P9), the unsubstituted scaffold (P0), and the lowest-affinity ligand (O5). The RNA backbone is shown in tubes, while RNA nucleotides directly interacting with the ligands are displayed in licorice and labeled. Colored dashed lines indicate RNA-ligand interactions, with green, red, and blue representing lipophilic, cation–anion, and hydrogen bond interactions, respectively.

To further assess the reliability of the predicted binding poses under explicit-solvent conditions and realistic ion concentrations, we performed atomistic MD simulations for the three representative ligands. At both ionic strengths (52 and 150 mM KCl), the main binding modes remained largely stable throughout the simulations, with occasional unbinding events observed for the lowest affinity ligand. The resulting RMSD profiles qualitatively reflected the experimental affinity ranking, with higher-affinity ligands exhibiting greater binding-mode stability (Figure S18). Notably, simulations conducted at the experimental salt concentration (52 mM) showed slightly more consistent stability profiles, particularly for the highest-affinity ligand, while preserving the same qualitative behavior. Altogether, these results indicate that the docking-derived poses are robust within the explicit-solvent solvation environment and across different ionic strengths.

## Conclusion

In this study, we applied a comprehensive structure-based drug discovery (SBDD) pipeline to investigate how a congeneric series of diminazene derivatives interacts with the MALAT1 triple helix, a prototypical structured lncRNA with known druggability. Our approach integrated enhanced molecular dynamics simulations, pocket communication analysis, ensemble docking, and rescoring with both classical and RNA-specific methods. The simulations revealed two persistent and ligand-accessible binding regions (Site 1 and Site 2), while the pocket communication analysis highlighted a degree of dynamic coupling between RNA subregions, hinting at potential for allosteric modulation. Ensemble docking across the generated RNA conformational ensemble expanded the diversity of accessible binding poses, particularly within Site 1, and uncovered complementary binding subpockets depending on the docking tool used. Despite limitations, AutoDock achieved modest discrimination between high- and low-affinity ligands. Crucially, our analysis of clustered interaction patterns using fingeRNAt and principal component analysis offered a clearer view of structure–affinity relationships. One specific subpopulation of poses (Site 2, Cluster 3) showed clear trends in relation to experimental affinity, suggesting this as a potentially relevant binding mode. Nevertheless, several limitations emerged that mirror challenges consistently reported in the field. The accuracy of scoring functions, both general-purpose and RNA-specific, including machine learning–based approaches, proved insufficient for reliably predicting binding affinities across structurally similar ligands, consistently with the general expectation for protein targets. In this respect, the known sensitivity of scoring functions to target conformations and the inherent difficulty in accounting for ligand and target flexibility become even more critical. Overall, our results reinforce prior observations that accurately generating and evaluating docking poses for RNA targets remains an open challenge, further complicated by the lack of high-resolution experimental binding affinity data for nucleic acid-ligand complexes. These data gaps hinder the development, training, and validation of more robust predictive models. In this context, it is important to note that the results obtained are inherently dependent on the docking software and scoring functions employed. We therefore expect that performance may change, and potentially improve, through the application of post-docking strategies such as MD-based relaxation of selected poses and more advanced rescoring approaches, whose systematic assessment was beyond the scope of the present work. In particular, MD-based refinement could be instrumental in explicitly accounting for solvent and ionic effects, as well as in elucidating the role of water and ions in stabilizing specific RNA conformations or ligand binding modes, which are challenging to capture at the docking stage.^60^ On the other hand, higher-level rescoring strategies may enable a more accurate estimation of binding affinities by partially mitigating the limitations associated with semiflexible docking approximations.^107,108^ Notably, free-energy methods based on alchemical transformations have reached a mature and consolidated stage in the context of protein-targeted drug discovery, where they have proven effective in improving affinity ranking and guiding lead optimization. Encouragingly, recent benchmark studies suggest that these approaches can achieve overall promising performances also when applied to RNA targets.^50,109,110^ While their routine application to RNA-focused drug discovery pipelines remains computationally demanding, continued methodological advances and increasing computational resources are expected to facilitate their broader adoption by the community. Finally, although the RNA model used here was structurally resolved and subjected to extensive conformational sampling, it may not fully capture the plasticity and environmental complexity present in cellular contexts. The absence of experimental confirmation for the predicted binding poses, combined with the limited chemical diversity of the ligand series, also limits the generalizability of our findings.

Altogether, our work underscores both the potential and the limitations of current SBDD strategies when applied to dynamic RNA targets. While advanced computational pipelines can extract useful structure–activity relationships and identify plausible binding modes, achieving predictive reliability remains out of reach. Progress in RNA-targeted drug discovery will require not only better scoring functions and more flexible modeling strategies but also broader access to high-quality experimental data and integrative validation frameworks.

## Supplementary Material



## Data Availability

All the materials to perform the MD simulations and the molecular docking calculations on the triple helix structure of the MALAT1 RNA, as well as to reproduce the analyses presented in this work is freely available in Zenodo with accession code 10.5281/zenodo.18872932. Specifically, necessary input files to perform both plain (i.e., unbiased) and Hamiltonian replica-exchange (HREX) MD simulations are provided together with the associated output trajectories. RNA and ligand structures for docking via AutoDock-GPU and rDock are also provided, as well as output data to reproduce the analyses and figures reported in this work. The Notebook_malat1.ipynb Jupyter Notebook illustrates how to perform all the analyses. The notebook can also be straightforwardly consulted at https://github.com/CompMedChemLab/project_malat1. Input files to perform plain MD simulations of the docking poses for three representative ligands (P9, P0, and O5), together with the corresponding output trajectories, are provided as well.

## Notes

Comparison of AutoDock grid box and rDock cavity mapping for the two binding regions; atom index transformations applied for minimum RMSD calculations; interpocket communication matrix obtained from Pocketron analysis; eRMSD timeseries for the two sites from conventional and HREX MD simulations; docking score distributions for all generated poses on representative RNA conformations of both sites, grouped by scoring function and docking software; evaluation of ligand-target binding using multiple scoring functions, highlighting top-ranked poses across RNA ensembles for both sites; analysis of RNA-ligand interactions via FingeRNAt; loadings of PC1 for different interaction features at both sites; PCA of the molecular fingerprints of all ligand poses for the two sites and resulting correlations between PC1 scores and relative binding affinity; RMSD analysis of compounds P9, P0, and O5 from MD simulations (PDF)

## Abbreviations

RNA: ribonucleic acid
MALAT1: metastasis-associated lung adenocarcinoma transcript 1
MD: molecular dynamics
HREX: Hamiltonian replica exchange
SBDD: structure-based drug discovery
